# Development of Taste Masked Oral Formulation of Ornidazole

**DOI:** 10.4103/0250-474X.65018

**Published:** 2010

**Authors:** Shishu, Kamalpreet, V. R. Kapoor

**Affiliations:** University Institute of Pharmaceutical Sciences, Panjab University, Chandigarh-160 014, India

**Keywords:** Eudragit E-100, fast disintegrating tablets, microspheres, ornidazole, taste masking

## Abstract

Taste masked microspheres of ornidazole were prepared using amino alkyl methacrylate copolymers (Eudragit E-100) by solvent evaporation technique. Taste assessment of these microspheres was done by both spectrophotometric taste evaluation technique and panel testing. Compressed tablets of taste masked ornidazole microspheres which rapidly disintegrated in the oral cavity were prepared using microcrystalline cellulose as directly compressible filler and sodium starch glycolate as a super-disintegrant. These were subsequently evaluated for various pharmacopoeial tests, drug release, and disintegration time in the oral cavity. Sensory taste evaluation was carried by panel testing in 20 healthy human volunteers. Results indicate successful formulation of oral fast disintegrating tablets which disintegrated in the oral cavity in about 30 s and possessed good taste.

Palatability of oral medications plays an important role in achieving compliance particularly in pediatric patients as most drugs taste bitter. Any pharmaceutical formulation with a pleasing taste and good flavor would definitely be preferred and translate into better compliance and therapeutic benefits. Oral administration of bitter drugs, using novel solid dosage forms essentially require an acceptable degree of taste masking. In the recent years, enormous development in taste masking technologies has given rise to novel strategies such as child friendly fast dissolving dosage forms, chewable tablets and taste masked suspensions. Amongst these dosage forms the fast disintegrating tablets (FDTs) have received ever-increasing popularity and acceptability during the last decade, and this field has become a rapidly growing area in pharmaceutical industry. When introduced into the mouth, these tablets dissolve or disintegrate in the mouth in the absence of additional water for easy administration of active pharmaceutical ingredients[1,2]. For achieving taste inhibition various chemical and physical methods that prevent the drug substance from interaction with taste buds are reported in the literature[3–5].

Ornidazole is widely used antiprotozoal drug. One of the major formulation problems with this drug is the bitter taste which gives rise to patient compliance problems when it is given as conventional dosage forms. In the present study taste masking of ornidazole was achieved by preparing taste masked microspheres using a pH-sensitive polymer, Eudragit E-100. These microspheres were then formulated into FDTs using the technique of super-disintegrant addition.

## MATERIALS AND METHODS

Ornidazole was procured from Sai Bliss Drug and Pharmaceuticals, Karnal, India. Eudragit E-100 was received as gift sample from Röhm Pharma, Darmstadt, Germany and Sodium starch glycolate was a gift from Kemwell Pvt. Ltd., Bangalore, India. Microcrystalline cellulose (Avicel-102) was received as gift sample from Panacea Biotec., Lalru, India. Lactose was procured from S.D. Fine Chem., Mumbai, India and D- mannitol GR from Loba Chemie Pvt. Ltd., Mumbai, India. Other tablet excipients and chemicals used were of AR grade.

### Preparation and evaluation of taste-masked microspheres:

Microspheres were prepared by solvent evaporation technique with necessary modifications[6,7]. Accurately weighed but varying amounts of Eudragit E-100 were dissolved in 10 ml of acetone over a cyclomixer and 250 mg of accurately weighed ornidazole was added in the polymer solution. Subsequently, 50 mg of magnesium stearate was added to the solution of polymer and drug in acetone. This organic phase was poured drop wise to 25 ml of 1:1 mixture of light and heavy liquid paraffin with vigorous stirring over a mechanical stirrer (M/s Remi motors, Mumbai-53). High stirring rates of approximately 6000 rpm were employed to obtain microspheres of smaller size. Stirring was continued for 3 h. Then 20 ml of hexane was added to the stirred contents. The suspension was filtered and microspheres were washed thrice with hexane, 10 ml each, to remove any adhering liquid paraffin from the surface. Finally, several washings with distilled water were given to remove any unentrapped drug from the surface of the microspheres. This was followed by vacuum drying of the microspheres overnight at room temperature. Several batches of microspheres were prepared by varying drug-polymer ratio, keeping all other formulation factors constant. The drug:polymer ratio 1:5 produced tasteless microspheres with highest entrapment efficiency (92%) which was used for further studies.

Optical microscopy was used to study the particle size distribution of the microspheres. Surface characteristics of microspheres were studied by scanning electron microscope (SEM; M/s Jeol, Japan, model number JSM-6100). Samples were mounted onto the stubs using double-sided adhesive tape and then coated with gold palladium alloy (150-200A°) using fine coat ion sputter. The samples were subsequently examined under the scanning electron microscope. For determining entrapment efficiency, 10 mg of accurately weighed sample of microspheres was crushed in a glass mortar. To this powdered mass, 5 ml of dehydrated alcohol was added and the contents were mixed thoroughly. The mixture was then filtered into a test tube. The mortar was rinsed with another 5 ml of dehydrated alcohol and added to the filtrate. The filtrate was adequately diluted with phosphate buffer pH 6.8 and analyzed spectrophotometrically at λ_max_ 320 nm using buffer as the blank. Entrapment efficiency of various samples were calculated using the formula, Entrapment efficiency = (weight of incorporated drug in mg/weight of drug used for microspheres preparation in mg)×100. The taste of microspheres was evaluated using spectrophotometric taste evaluation technique[8] and by panel testing[9]. The method to evaluate the taste masking of drug spectrophotometrically was based on the bitterness threshold concentration of any drug. The threshold value of bitter taste for the drug was first judged by a sensory test on human volunteers. Subsequently, a known quantity of the supposedly taste-masked formulation was mixed with 10 ml of phosphate buffer pH 6.8 in a 10 ml injection syringe by revolving the syringe end to end, five times in 30 s. The test medium was then filtered through a membrane filter, followed by spectrophotometric determination of the concentration of drug in the filtrate. This concentration of drug was compared with the bitterness threshold concentration obtained by panel testing.

### Formulation of fast disintegrating tablets:

Fast disintegrating tablets were prepared using super-disintegrant addition technique. Different ratios of microcrystalline cellulose (MCC) and sodium starch glycolate (SSG) as shown in Table 1 were used. Accurately weighed microspheres, microcrystalline cellulose, sodium starch glycolate, mannitol and lactose were blended manually in a polyethylene bag for about 10-15 min. Then menthol and magnesium stearate were added and mixed for further 2 min and compressed into tablets using round double concave punches of dimension 15 mm. The ratio of SSG/MCC giving the minimum disintegration time along with optimum hardness was selected for the preparation of the final batch of tablets (Table 2). Similarly control tablets were compressed using pure drug instead of taste-masked microspheres. The fracture strength was measured with Monsanto tablet hardness tester[10].

**TABLE 1:** EFFECT OF SSG AND MCC RATIO ON VARIOUS PHYSICAL PROPERTIES OF ORNIDAZOLE FDTS

Formulation code	SSG: MCC	Disintegration time (s) (n=6)	Tablet strength (kg/cm^2^)
O -1	1:0.5	7±1.26	0.5
O -2	1:1.0	17±1.36	0.5
O -3	1:2.0	37±1.12	1.5
O -4	1:3.0	53±2.34	2.0
O -5	1:4.0	76±2.94	2.5
O -6*	1:5.0	108±1.54	3.0
O -7	1:6.0	146±2.64	3.5
O -8	1:7.0	181±1.35	4.5
O -9	1:8.0	196±1.46	5.5
O -10	1:9.0	205±1.56	9.0

* O-6 represents optimum ratio of SSG: MCC for preparation of FDTs

**TABLE 2:** FORMULA FOR TASTE-MASKED ORNIDAZOLE FDTS

Ingredient(s)	Quantitiy (mg/tablet)
Microspheres	650.0
MCC	102.0
SSG	20.0
Menthol	5.0
Magnesium stearate	5.0
Mannitol	170.0

To test friability pre-weighed tablets were placed in a Roche Friability tester which was rotated for 4 min at 25 rpm. Then the tablets were weighed and the loss in weight (%) was calculated[11]. To measure tablet wetting time a piece of paper tissue folded twice was placed in a small culture dish (i.d.= 6.5 cm) containing 6 ml of water, a pre-weighed tablet was placed on the paper, and the time for complete wetting was measured. The wetted tablet was then weighed and water absorption ratio, R, was determined according to the equation: R=100(W_a_–W_b_)/W_bv_, where W_a_ and W_b_ are the weight after and before water absorption, respectively[12].

*In vitro* dissolution studies were carried out using USP XXIII Dissolution apparatus II (paddle type) for six tablets. Each of the six rapidly disintegrating tablets containing microspheres were placed separately in dissolution beakers containing dissolution medium (900 ml) which was rotated at a speed of 50 rpm by means of a paddle. A temperature of 37±0.5° was maintained throughout the study. The release profile was studied both in phosphate buffer at pH 6.8 and hydrochloric acid buffer pH 1.2. Samples were analyzed spectrophotometrically at λ_max_ 320 nm using buffer as the blank[13].

For the measurement of *in vitro* disintegration time a modified dissolution apparatus (paddle type) was used. Nine hundred milliliters of water maintained at 37±0.5° and stirred with a paddle at 100 rpm was used as the disintegration fluid. Six tablets were placed individually in six sinkers and disintegration time was determined at the point at which all the tablet disintegrated completely and passed through the screen of the sinker[12,13].

Disintegration time in the oral cavity was also determined. Six healthy volunteers, from whom informed consent was first obtained, randomly took one tablet and the time required for complete disintegration of the tablet in the mouth, without biting and without drinking water, was measured[12].

The taste evaluation was done by panel testing. For this, 20 healthy human volunteers, of either sex, in the age group of 20-30 years were selected out of 31 volunteers based on the 6-n-propylthiouracil (Prop) taste sensitivity test[14]. Taste threshold for all the volunteers was determined by making a range of dilutions of propylthiouracil. The non-tasters and super-tasters were rejected. Then a selected panel of 20 healthy human volunteers was requested to taste the taste-masked FDTs by keeping in the mouth till they disintegrated and rank it on a scale of perception ranging from 0-5 (0=good, 1=tasteless, 2=slightly bitter, 3=bitter, 4=very bitter, 5=awful). For comparison, FDTs of pure ornidazole were also subjected to taste evaluation by the same panel and the results were compared[9].

## RESULTS AND DISCUSSION

**Fig. 1:**
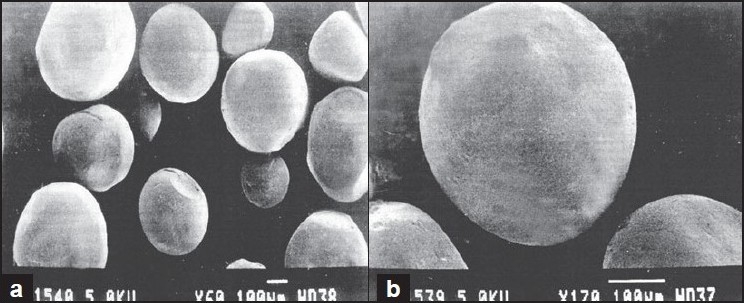
Scanning electron photomicrograph of ornidazole microspheres Scanning electron photomicrograph of ornidazole microspheres at (a) magnification of 60X. (b) magnifi cation of 170X

Particle size distribution of microspheres was observed to be log normal and mean particle size of microspheres was found to be 150.075±23.59 µm. It was observed from the photomicrographs that the surface of microspheres was free of any adhering drug and these were spherical in shape (figs. 1a and 1b). The minimum concentration among a range of dilutions of a substance at which a volunteer just starts feeling the bitter taste is known as ‘threshold concentration’. The threshold bitterness concentration of ornidazole as determined by the panel was found to be 70 µg/ml.

The results obtained by spectrophotometric method of analysis for taste of taste-masked microspheres showed that only 16±1.0 µg/ml of drug (less than ¼ th of the threshold bitterness concentration) was released in phosphate buffer pH 6.8 after 30 s of contact time. Also, sensory evaluation for taste by panel confirmed the tasteless characteristics of the microspheres as none of the subjects felt bitter taste even after keeping them in mouth for 20-30 s. All the 20 volunteers reported these as tasteless on the perception scale. Hence this technique of taste-masking using a pH-sensitive polymer was found to be effective in masking the bitter taste of the drug.

Formulation of taste-masked microspheres into tablets faces the challenge that the original properties of microparticles should be retained during compression. This was attained by keeping the compression pressure low and using the direct compression procedure instead of wet granulation, thus avoiding lengthy granulation steps and exposure to various solvents used in wet granulation. MCC (20-90%) was used as a directly compressible binder and SSG (1-10%) as the super-disintegrant. Lactose and menthol were added to give the tablets a more palatable feel. Magnesium stearate (1%) was used as a lubricant and mannitol as a diluent.

The aim of our study was to prepare tablets that not only disintegrated rapidly in the oral cavity but also had acceptable hardness for easy handling. Therefore, various ratios of the super-disintegrant, SSG and the binder, MCC were investigated and the ratio that gave the minimum disintegrating time along with acceptable hardness was selected for the formulation of the final batch of tablets. Although the formulations with ratios 1:0.5 and 1:1 of SSG:MCC disintegrated very fast in approximately 7 and 17 s, respectively, the hardness of the tablets was also very low (0.5 kg/cm^2^), making the tablets very soft. Formulation O-6 gave the disintegration time of 108±1.54 s with acceptable hardness value. Therefore, this formulation was selected for preparing the final batch of tablets (Table 1).

A linear correlation between the disintegration time and wetting time of the FDTs was observed, suggesting that wetting is an important step for the disintegration process (y = 2.0112x and R^2^ = 0.9989).

**Fig. 2:**
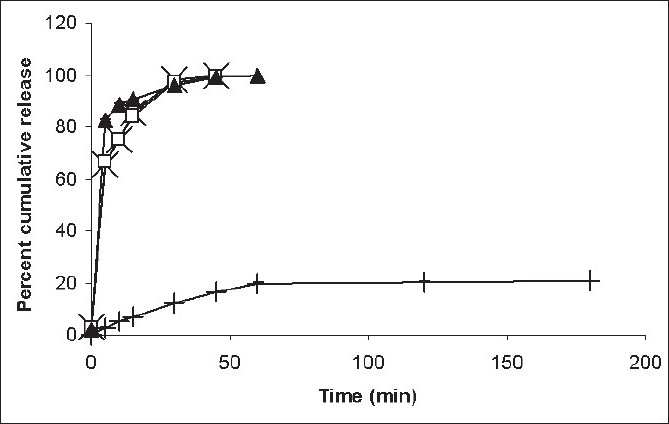
Comparison of *in vitro* release profiles of different formulations of ornidazole. Plot showing percent cumulative release from pure drug (–×–) and FDT (–□–) in HCl buffer at pH 1.2; pure drug (–▲–) and FDT (–+–) in phosphate buffer at pH 6.8. Mean ±SD, n=6

Fig. 2 shows the dissolution profiles of taste-masked and control (pure drug) FDTs, at two different pH values. It was found that there exists a marked difference in the dissolution profiles of pure drug and encapsulated drug FDTs at pH 6.8. As high as 90% of drug was released from the control in 15 min while only 7% was released from the FDTs of microspheres. These results show the pH-sensitive nature of Eudragit E-100 i.e. it does not dissolve at pH above 5. This also suggests that sufficient taste masking has been achieved and the bitter taste of the drug will not be perceived while the tablet is in the mouth.

As Eudragit E-100 is soluble at pH below 5, hydrochloric acid buffer pH 1.2 simulating the gastric fluid was chosen as the second medium. It was found that about 85% of ornidazole was released from control tablets and 84% was released from the FDTs of taste masked ornidazole after 15 min of dissolution, suggesting that the drug would be easily released from the microspheres in the acidic pH of stomach. Further, the release profile of the drug from the FDTs of taste masked microspheres and of plain drug was observed to be comparable and superimposable at pH 1.2, indicating that the drug will be released at approximately the same rate and to the same extent in the stomach. Thus showing that taste-masking of the drug with the pH-sensitive polymer would not affect the release of the drug in the stomach.

The hardness[15] and friability[16] of the ornidazole FDTs were found to be satisfactory and was within the limits specified for fast disintegrating tablets. The wetting time was found to be 52.13±1.71 s and the water absorption ratio was 18.34±0.77. The disintegration time of the FDTs in the external medium (108 s) was found to be within the limits (i.e. within 180 s)[16]. Also disintegration time in the oral cavity of the volunteers was 29 s (i.e. within limits of 5-90 s) as reported for fast disintegrating tablets[16]. Thus showing that fast disintegration has been successfully achieved (Table 3).

**TABLE 3:** PHYSICAL PROPERTIES OF TASTE-MASKED ORNIDAZOLE FDTS

Parameter	Value
Weight (mg), n=20	952.25±1.60
Diameter (cm), n=6	1.50±0.004
Thickness (cm), n=6	0.75±0.01
Hardness (kg/cm), n=6	3.00±0.20
Friability (%), n=6	0.09%
Wetting time (s), n=3	52.13±1.71
Water absorption ratio, n=3	18.34±0.77
*In vitro* disintegration time (s), n=6	108±1.53
Oral disintegration time (s), n=6	29.31±1.17
Drug content (mg), n=3	123.63±1.16

All the 20 volunteers reported the ornidazole microspheres as tasteless and 19 volunteers reported the FDTs of ornidazole microspheres as good and 1 reported it as tasteless. This suggests that the bitter taste of ornidazole in the microspheres continued to be masked on being compressed into tablets. The FDTs of plain ornidazole were rated as slightly bitter to very bitter by the panel and the FDTs of microspheres were rated as good by 95% and tasteless by 5% of the panel. Moreover, all the volunteers experienced a good mouth feel of the FDTs as these dissolved in the mouth with minimum of grit.

To conclude, fast disintegrating tablets of ornidazole were successfully prepared by the technique of super-disintegrant addition. Also, as a prerequisite, effective taste-masking was achieved for ornidazole by preparation of taste-masked microspheres using pH sensitive polymer. These studies suggest that these patient compliant dosage forms after complete stability evaluation may be successfully commercialized.
